# Prevalence of oropharyngeal dysphagia and risk of mortality among hospitalized COVID-19 patients: A meta-analysis

**DOI:** 10.7189/jogh.12.05058

**Published:** 2022-12-29

**Authors:** Chi-Li Lee, Garry Huang, Kondwani Joseph Banda, Yu-Hao Chu, Hsiu-Ju Jen, Hsin Chu, Doresses Liu, Li-Chung Pien, Ruey Chen, Kuei-Ru Chou

**Affiliations:** 1 Division of Intensive Care Medicine, Department of Internal Medicine, Yuan's General Hospital, Kaohsiung, Taiwan; 2 School of Nursing, College of Nursing, Taipei Medical University, Taipei, Taiwan; 3 School of Health Care Administration, College of Management, Taipei Medical University, Taipei, Taiwan; 4 Australasian College of Paramedicine, Australia; 5 Australian Institute of Project Management, Australia; 6 Endoscopy Unit, Surgery Department, Kamuzu Central Hospital, Lilongwe, Malawi; 7 School of Nutrition and Health Sciences, College of Nutrition, Taipei Medical University; 8 Department of Nursing, Taipei Medical University-Shuang Ho Hospital, New Taipei, Taiwan; 9 Institute of Aerospace and Undersea Medicine, School of Medicine, National Defense Medical Center, Taipei, Taiwan; 10 Department of Neurology, Tri-Service General Hospital, National Defense Medical Center, Taipei, Taiwan; 11 Department of Nursing, Wan Fang Hospital, Taipei Medical University, Taipei, Taiwan; 12 Center for Nursing and Healthcare Research in Clinical Practice Application, Wan Fang Hospital, Taipei Medical University, Taipei, Taiwan; 13 Post-Baccalaureate Program in Nursing, College of Nursing, Taipei Medical University, Taipei, Taiwan; 14 Psychiatric Research Center, Wan Fang Hospital, Taipei Medical University, Taipei, Taiwan; 15 Psychiatric Research Center, Taipei Medical University Hospital, Taipei, Taiwan; 16 Neuroscience Research Center, Taipei Medical University, Taipei, Taiwan

## Abstract

**Background:**

Post-extubation and neurologic complications in COVID-19 patients have been shown to cause oropharyngeal dysphagia (OD). We performed the first meta-analysis to explore and estimate the pooled prevalence of OD, risk of mortality, and associated factors among hospitalized COVID-19 patients.

**Methods:**

We searched Scopus, PubMed, Embase, CINAHL, WHO COVID-19 database, and Web of Science for literature on dysphagia in COVID-19 patients. We used the generalized linear mixed model (GLMM) to determine the prevalence estimates of OD in the R software and the DerSimonian-Lard random-effects model in the Comprehensive Meta-Analysis software to explore the risk of mortality and associated factors of OD, presented as odds ratios (ORs) and corresponding 95% confidence intervals (CIs). We used Cochran's Q, τ^2^, and the *I*^2^ statistic to assess heterogeneity and conducted a moderator analysis to identify moderator variables.

**Results:**

We included eighteen studies with a total of 2055 participants from the 910 studies retrieved from electronic databases. The prevalence of OD among hospitalized COVID-19 patients was estimated at 35% (95% CI = 21-52; low certainty of evidence) associated with a high risk of mortality (OR = 6.41; 95% CI = 1.48-27.7; moderate certainty of evidence). Intubation (OR = 16.3; 95% CI = 7.10-37.3; high certainty of evidence), use of tracheostomies (OR = 8.09; 95% CI = 3.05-21.5; high certainty of evidence), and proning (OR = 4.97; 95% CI = 1.34-18.5; high certainty of evidence) among hospitalized COVID-19 patients were highly associated with developing OD. The prevalence of OD was higher among hospitalized COVID-19 patients who were admitted in intensive care units (ICU), intubated, and mechanically ventilated.

**Conclusions:**

The prevalence of OD among hospitalized COVID-19 patients is estimated at 35% associated with a high risk of mortality. OD assessment among hospitalized COVID-19 patients who are managed in an ICU, prone position, intubated, and mechanical ventilated deserves more attention.

**Registration:**

PROSPERO CRD42022337597

Globally, over 500 million coronavirus disease 2019 (COVID-19) cases have been reported and confirmed, with six million deaths as of June, 2022 [1]. COVID-19 has been associated with neuropsychiatric, cardiovascular, hematologic, musculoskeletal, renal, dermatologic, endocrine, pulmonary, and digestive complications [2,3]. Growing evidence revealed oropharyngeal dysphagia (OD) as one of the digestive complications among hospitalized COVID-19 patients. Moreover, OD has been shown to be highly correlated with a risk of aspiration pneumonia, malnutrition, and mortality in acute stroke and older adults without COVID-19 [4,5]. Exploring and understanding the prevalence of OD in COVID-19 patients would provide plausible evidence crucial for the management of COVID-19-associated OD in clinical settings.

Current evidence on COVID-19-associated OD has shown that neurologic complications (including myopathy and polyneuropathy) in COVID-19 patients are linked to damage of the swallowing neural network [6]. Additionally, COVID-19 virus’ invasion of the peripheral nerves causes anosmia, ageusia, and impaired oropharyngeal sensory function, leading to swallowing impairment in COVID-19 patients [6]. Management of COVID-19 patients with endotracheal intubation, proning, and mechanical ventilation in intensive care units (ICU) and COVID-19 wards due to severe acute respiratory distress syndrome (ARDS) has been a crucial COVID-19 management element [7]. As such, prolonged intubation and mechanical ventilation have been shown to cause laryngeal oedema, granulations, and glottis stenosis, leading to delayed oral intake initiation, prolonged tube feeding, aspiration, increased morbidity, and mortality among hospitalized COVID-19 patients [7]. However, evidence on the pooled prevalence of OD among hospitalized COVID-19 patients is lacking and no meta-analysis study has explored and estimated the overall prevalence of OD. As COVID-19 remains a major public health concern, identifying the nature and extent of OD associated with COVID-19 neurological involvement would be crucial for the prevention and better management of aspiration pneumonia and malnutrition to reduce mortality in this population. To address this gap, we performed the first meta-analysis to explore and estimate the prevalence of OD, risk of mortality, and its associated participants, disease, and methodological factors in hospitalized COVID-19 patients.

## METHODS

### Search strategy

The conduct and reporting of this meta-analysis followed the Meta-Analysis of Observational Studies in Epidemiology (MOOSE) and updated 2020 Preferred Reporting Items for Systematic Reviews and Meta-Analyses (PRISMA) guidelines with PROSPERO registration number: CRD42022337597 [8]. We comprehensively searched the WHO COVID-19 database, Embase, PubMed, Cochrane Library, Web of Science, and Scopus from January 2020 until June 2022 using the following keywords: (prevalence OR incidence OR epidemiology OR rate OR rates OR number OR proportion OR probability OR event) AND (dysphagia OR swallowing disorder OR deglutition disorder OR oropharyngeal dysphagia) AND (COVID-19 or covid-19 OR Corona virus OR SARS-COV 2) (detailed search strategy is available in Table S1 in the **Online Supplementary Document**). A manual search of references was performed in relevant previous observational studies and systematic reviews for additional potential eligible studies, which were then searched in Google to identify their published full text. Additionally, we contacted corresponding authors to provide missing data.

### Study selection

**Figure 1:**
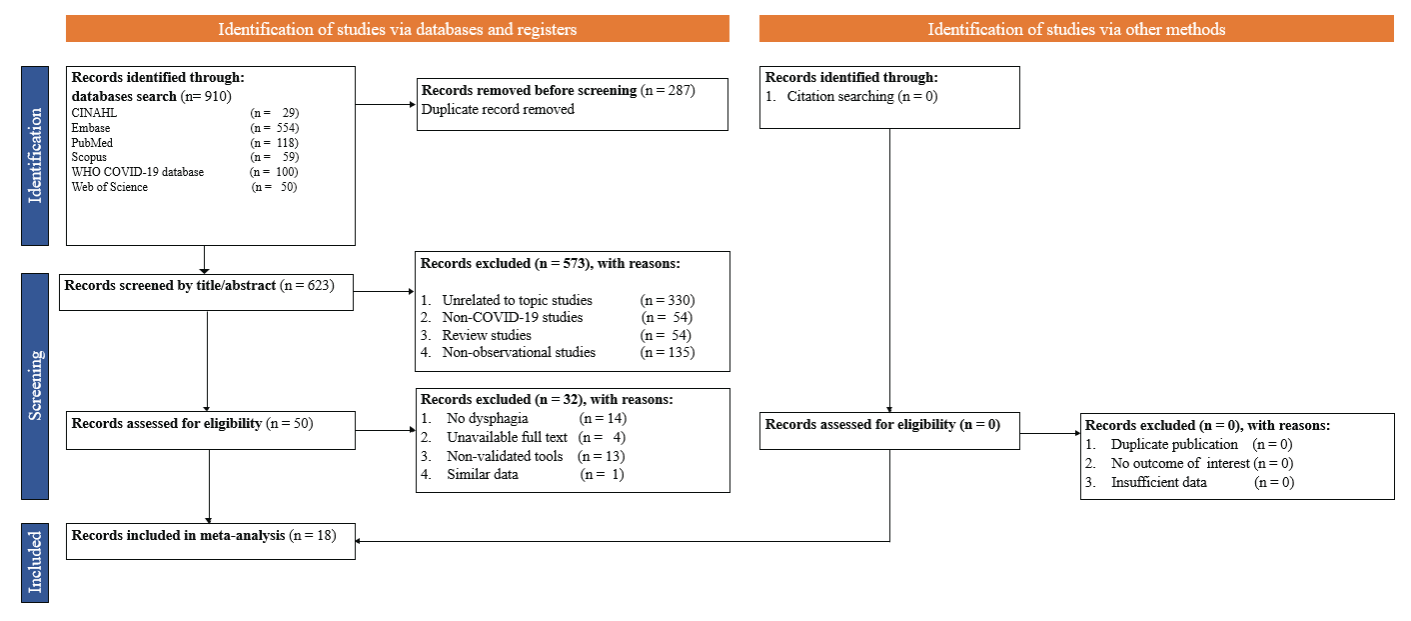
Flowchart for study selection.

The study inclusion criteria without any language restrictions were: 1) population: adults ≥18 years old with confirmed COVID-19, 2) exposure: OD, 3) comparator: no OD, 4) outcome: epidemiology, incidence, or prevalence, 5) study design: retrospective and prospective cohort studies, and 6) validated OD assessment tool. The exclusion criteria were: 1) non-COVID-19 studies, 2) systematic review or meta-analysis studies, 3) studies using non-validated assessment tools, 4) duplicate studies, 5) studies unrelated to the topic, and 6) non-observational studies (**Figure 1**).

### Data extraction and study outcomes

CLL and GH extracted the following data from the included studies using standard data extraction forms: author, year of publication, age, study period, sample size, setting (ICU and non-ICU), gender, study design (retrospective and prospective cohort), country, dysphagia assessment, prevalence (number and %), and associated moderator factors (intubation, ventilation, and proning status and neurologic comorbidities).

The primary outcome was pooled prevalence of OD using validated invasive, objective, and subjective clinical assessment tools in hospitalized COVID-19 patients. The secondary outcomes were risk of mortality and associated factors, including gender, intubation, tracheostomy use, proning, mechanical ventilation, and comorbidities (including respiratory diseases, hypertension, diabetes mellitus, and neurological diseases) (**Table 1** and **2**).

**Table 1:** Demographic characteristics for hospitalized COVID-19 patients with oropharyngeal dysphagia

Author (year)	Country	Mean age (SD)	Sample (N)	Gender (M/F)	Study design	Study period
Martin-Martinez et al., (2021) [6]	Spain	69.3 ± 17.5	205	98/107	Prospective cohort	April to July, 2020
Archer et al., (2021) [9]	UK	56.8 ± 16.7	164	104/60	Prospective cohort	April to May, 2020
Ceruti et al., (2021) [10]	Switzerland	61 ± 12	31	25/6	Retrospective cohort	March to April, 2020
Clayton et al., (2021) [11]	Australia	65 ± 15.5	27	22/5	Prospective cohort	March 2020 to March 2021
Gonzalez Lindh et al., (2022) [12]	Sweden	61 ± 12	28	22/6	Prospective cohort	March to July, 2020
Grilli et al., (2022) [13]	Italy	51.3 ± 16.5	41	20/21	Prospective cohort	March to May, 2020
Lagier et al., (2021) [14]	Belgium	61.8 ± 7.8	21	14/7	Prospective cohort	2020
Laguna et al., (2021) [15]	Spain	60.4 ± 0.6	232	172/60	Retrospective cohort	March to May, 2020
Leis-Cofino et al., (2021) [16]	Spain	61.1 ± 3.6	40	24/16	Prospective cohort	March to April, 2020
Leis-Cofino et al., (2021a) [16]	Spain	67.8 ± 4.8	39	24/15	Prospective cohort	March to April, 2020
Lima et al., (2020) [17]	Brazil	53.4 ± 15.9	77	NA	Prospective cohort	2020
Marchese et al., (2022) [18]	Italy	57.0 ± 4.7	117	69/48	Prospective cohort	March to July, 2020
Olezene et al., (2021) [19]	USA	59.5 ± 4.3	29	20/9	Retrospective cohort	April to May, 2020
Regan et al., (2021) [20]	Ireland	66.5 ± 13.3	315	194/121	Prospective cohort	March to June, 2020
Reyes-Torres et al., (2021) [21]	Mexico	54.0 ± 12.0	112	92/20	Prospective cohort	September to December, 2020
Rouhani et al., (2021) [22]	UK	55.8 ± 11.3	41	28/13	Prospective cohort	April to May, 2020
Webler et al., (2022) [23]	USA	65.9 ± 13.0	40	29/11	Retrospective cohort	April to August, 2020
Yamada et al., (2021) [24]	Japan	52.7 ± 19.0	456	284/172	Retrospective cohort	January to September, 2020
Yilmaz et al., (2021) [25]	Turkey	65.1	40	NA	Retrospective cohort	March 2020 to February, 2021

F – female, M – male, N – total sample size, NA – not available, SD – standard deviation, UK – United Kingdom, USA – United States of America

**Table 2:** Disease characteristics for hospitalized COVID-19 patients with oropharyngeal dysphagia

Author (year)	Setting	Dysphagia Assessment	Prevalence (n)	Prevalence (%)	Associated moderator factors
Martin-Martinez et al., (2021) [6]	COVID-19 ward	V-VST	106	51.7	Intubation: no; ventilation: no; proning status: no; neurologic comorbidities: yes
Archer et al., (2021) [9]	Rehabilitation ward	FOIS	53	32.3	Intubation: yes; ventilation: yes; proning status: yes; neurologic comorbidities: yes
Ceruti et al., (2021) [10]	ICU	GUSS	17	15.3	Intubation: yes; ventilation: yes; proning status: yes; neurologic comorbidities: no
Clayton et al., (2021) [11]	ICU	FOIS	25	93.0	Intubation: yes; ventilation: yes; proning status: yes; neurologic comorbidities: no
Gonzalez Lindh et al., (2022) [12]	ICU	FOIS	20	71.0	Intubation: yes; ventilation: yes; proning status: yes; neurologic comorbidities: no
Grilli et al., (2022) [13]	COVID-19 department	V-VST	8	19.5	Intubation: no; ventilation: no; proning status: no; neurologic comorbidities: yes
Lagier et al., (2021) [14]	ICU	VFSS	19	90.5	Intubation: yes; ventilation: yes; proning status: yes; neurologic comorbidities: yes
Laguna et al., (2021) [15]	ICU	V-VST	27	11.6	Intubation: yes; ventilation: yes; proning status: yes; neurologic comorbidities: no
Leis-Cofino et al., (2021) [16]	ICU	EAT-10	7	17.5	Intubation: yes; ventilation: yes; proning status: yes; neurologic comorbidities: no
Leis-Cofino et al., (2021a) [16]	COVID-19 ward	EAT-10	1	2.6	Intubation: no; ventilation: no; proning status: no; neurologic comorbidities: no
Lima et al., (2020) [17]	ICU	ASHA-NOMS	14	18.2	Intubation: yes; ventilation: yes; proning status: yes; neurologic comorbidities: no
Marchese et al., (2022) [18]	COVID-19 ward	EAT-10	8	6.8	Intubation: yes; ventilation: yes; proning status: no; neurologic comorbidities: no
Olezene et al., (2021) [19]	Rehabilitation ward	ASHA-NOMS	25	86.2	Intubation: yes, ventilation: yes, proning status: no, neurologic comorbidities: no
Regan et al., (2021) [20]	Rehabilitation wards	FOIS	81	25.8	Intubation: yes; ventilation: yes; proning status: yes; neurologic comorbidities: yes
Reyes-Torres et al., (2021) [21]	ICU	V-VST	46	41.1	Intubation: yes; ventilation: yes; proning status: yes; neurologic comorbidities: no
Rouhani et al., (2021) [22]	COVID-19 ward	EAT-10	12	29.3	Intubation: yes; ventilation: yes; proning status: no; neurologic comorbidities: no
Webler et al., (2022) [23]	Rehabilitation ward	FOIS	8	20.0	Intubation: yes; ventilation: yes; proning status: no; neurologic comorbidities: yes
Yamada et al., (2021) [24]	COVID-19 ward	FILS	40	20.1	intubation: yes; ventilation: yes; proning status: yes; neurologic comorbidities: yes
Yilmaz et al., (2021) [25]	ICU	GUSS	24	60.0	Intubation: yes; ventilation: yes; proning status: yes; neurologic comorbidities: yes

ASHA-NOMS – American Speech Language Hearing Association’s National Outcomes Measurement System, COVID-19 – coronavirus disease 2019, EAT-10 – Eat Assessment Tool-10, FILS – Food Intake Level Scale, FOIS – Functional Oral Intake Scale, GUSS – Gugging Swallowing Screen, ICU – intensive care unit, n – dysphagia cases, VFSS – videofluoroscopic swallowing study, V-VST – volume-viscosity swallow test

### Quality assessment and certainty of evidence of included studies

We examined the quality of the included studies using Hoy’s Risk of Bias assessment tool [26]. The selection and non-response bias focus on the external validity of the study domains. The measurement bias and bias related to the analysis focus on the internal validity of the study domains. The score for each item for the domains is 1 for low risk and 0 for high risk, with the overall quality ranking graded as 9-10 for a high-quality study, 8-7 for a moderate quality study, and 0-6 for a poor quality study (Table S2 in the **Online Supplementary Document**). A third expert reviewer (KRC) resolved and addressed discrepancies between CLL and GH through discussions.

The certainty of evidence was assessed by examining the outcome effect estimates among the included studies using the GradePro criteria [27]. The GradePro criteria for certainty of evidence has five domains – publication bias, indirectness, risk of bias, imprecision, and inconsistency rated as serious and not serious. The overall certainty of evidence for each study outcome is rated as 1) high, 2) moderate, 3) low, and 4) very low (Table S3 in the **Online Supplementary Document**).

### Statistical methods

We determined the pooled estimates of OD in hospitalized COVID-19 patients using a generalized linear mixed model (GLMM) in R software [28,29]. GLMM accounts for within-study variations when combining proportions in prevalence meta-analysis and includes studies with small sample sizes and rare events. It uses binomial likelihoods for each included study with no data transformations on prevalence proportions and requires no corrections for zero counts in the estimation of proportions. Furthermore, specific link functions are used in GLMM to transform latent true prevalence proportions. We inspected the prevalence estimates on the funnel plot and Egger’s regression method to determine publication bias [30]. We used heterogeneity assessment to explore study variations in the OD prevalence estimates andX^2^-based test using Cochran's Q (*P* < 0.10), τ^2^, and *I*^2^ statistics to assess heterogeneity [28,31] with low (0%-25%), moderate (25%-75%), and high (≥75%) heterogeneity.

We assessed the associated outcome and factors of OD using the DerSimonian-Lard random and fixed-effects models in the Comprehensive Meta-Analysis (CMA) software (version 2.0) [32]. CMA determined the pooled odds ratios (ORs) for OD associated outcome and factors using the inverse variance-weighted mean of the logarithm of OR with 95% confidence intervals (CIs). OR was the effect estimate measure that explained the association between OD with risk of mortality and associated factors, including gender, intubation, tracheostomy use, proning, mechanical ventilation, and comorbidities (including respiratory diseases, hypertension, diabetes mellitus, and neurological diseases).

### Moderator analysis

We conducted a moderator analysis through meta-regression and sub-group analysis using pre-specified variables among the included studies [32,33]. We conducted meta-regression models for mean age and gender, including male and female percentages for continuous variables. We conducted sub-group analyses for categorical variables, including ventilation status (mechanical ventilated, non-mechanical ventilated, and combined), clinical assessment methods (invasive, objective, and subjective), continent (Asia, America, and Europe), study design (retrospective and prospective), intubation status (intubated, non-intubated, and combined), study quality (high, moderate and poor), study type (document review and primary study), proning status (yes and no), and neurologic comorbidities (yes and no).

### Ethical approval

No ethical approval was required for this study.

## RESULTS

We identified and included 18 studies [6,9-25] published between 2020 and 2022 from 910 studies retrieved from WHO COVID-19 database, PubMed, Scopus, CINAHL, Embase, Psych Info, and Web of Science (**Figure 1**). We included a total of 2055 participants (64% males and 36% females) in our meta-analysis. Four of the included studies were done in North and South America, three in Asia, and 11 in Europe. Regarding study setting, eight were conducted in ICU and ten in non-ICU including rehabilitation and COVID-19 wards. Regarding study design, a cross-sectional design was used in six studies and a prospective cohort design in 12. Among these studies, six were of high quality, nine were of moderate quality, and three were of poor quality. In the studies, OD was assessed by Videofluoscopic Swallowing Study (VFSS), Gugging Swallowing Screen (GUSS), Volume Viscosity Swallow Test (V-VST), Functional Oral Intake Scale (FOIS), Eat Assessment Tool-10 (EAT-10), American Speech Language Hearing Association’s (ASHA) National Outcomes Measurement System (NOMS) (ASHA-NOMS), and Food Intake Level Scale (FILS). The prevalence of OD ranged from 2.6% to 93.0% (**Table 1** and **Table 2**).

### Prevalence of OD and risk of mortality among hospitalized COVID-19 patients

**Figure 2:**
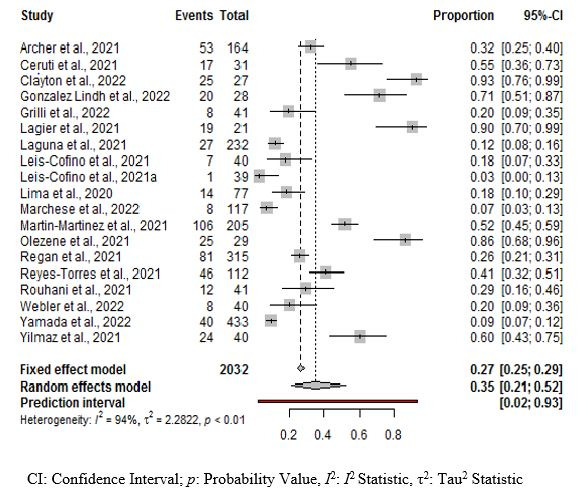
Prevalence of oropharyngeal dysphagia among hospitalized COVID-19 patients.

The pooled prevalence of OD among for hospitalized COVID-19 patients was estimated at 35% (95% CI = 21, 52; low certainty of evidence). There was statistical heterogeneity among the included studies with Q = 431.64, τ^2^ = 2.2822 and *I*^2^ = 94% (*P* = 0.01) **(****Figure 2****)**. We found some evidence of publication bias with the Egger regression (co-efficient = 8.75, t-value = 4.01; *P* = 0.01, and the funnel plot showing asymmetry of the prevalence estimates) among the included studies (Figure S1 in the **Online Supplementary Document**). The prevalence of OD was higher in COVID-19 patients admitted to the ICU estimated at 53% compared to 23% for those admitted in rehabilitation and COVID-19 wards. Among the continents (*P* = 0.62), the prevalence of OD was 55% for Asia, 40% for America, and 29% for Europe **(****Table 3****)**. Among the included studies, three studies with four estimates assessed the risk of mortality among hospitalized COVID-19 patients. Hospitalized COVID-19 patients with OD were more likely to die compared to hospitalized COVID-19 patients with no OD (OR = 6.41; 95% CI = 1.48, 27.7; moderate certainty of evidence) **(****Table 4****)**.

**Table 3:** Moderator analysis

Variables	*P*-value	n	Prevalence (95% CI)
**Participants’ characteristics**			
**Age**	0.29	19	7% (-6, 20)
**Male percentage**	0.12	19	3% (-1, 7)
**Female percentage**	0.29	19	-3% (-9, 3)
**Continent**	0.62	19	
Asia		3	55% (11, 92)
America		4	40% (16, 71)
Europe		12	29% (16, 48)
**Setting**	0.07	19	
ICU		9	53% (28, 74)
Non-ICU		10	23% (12, 41)
**Ventilation status**	<0.01	19	
Mechanical ventilated		7	71% (47, 87)
Non-mechanical ventilated		6	25% (13, 42)
Combined		6	15% (10, 24)
**Intubation status**	<0.01	19	
Post-intubated		9	63% (42, 80)
Non-intubated		6	18% (4, 55)
Combined		4	16% (10, 24)
**Proning status**	0.29	19	
Yes		12	42% (24, 62)
No		7	24% (9, 52)
**Neurological comorbidities**	0.93	19	
Yes		8	36% (19, 57)
No		11	34% (15, 60)
**Methodological characteristics**			
**Assessment method**	<0.01	19	
Invasive method		1	90% (69, 98)
Non-invasive objective method		6	37% (22, 55)
Non-invasive subjective method		12	29% (14, 52)
**Study quality**	0.14	19	
Poor quality		3	55% (21, 85)
Moderate quality		9	43% (20, 70)
High quality		7	21% (11, 38)
**Study design**	0.95	19	
Retrospective cohort		6	36% (15, 65)
Prospective cohort		13	35% (18, 56)
**Study type**	0.80	19	
Document review		8	38% (15, 68)
Primary study		11	33% (19, 53)

CI – confidence interval, ICU – intensive care unit, n – number of studies

**Table 4:** Prevalence and associated outcome and factors of oropharyngeal dysphagia in COVID-19 patients

Variables	n	Prevalence (95% CI)	*P*-value
**Pooled prevalence**	18	35% (21-52)	<0.01
**Associated outcome (OR, 95% CI)**			
Mortality	3	6.4 (1.48-27.7)	0.01
**Associated factors**			
Intubation	2	16.3 (7.10-37.3)	<0.01
Tracheostomy use	2	8.1 (3.05-21.5)	<0.01
Proning	2	4.9 (1.34-18.5)	0.02
Mechanical ventilation	2	3.3 (0.30-36.7)	0.34
**Comorbidities**			
Respiratory disease	2	2.4 (0.52-11.1)	0.26
Hypertension	3	2.1 (0.45-9.58)	0.35
Diabetes mellitus	4	1.3 (0.62-2.66)	0.50
Neurological disease	2	1.3 (0.14-11.6)	0.83
**Participant characteristics**			
Male	4	0.7 (0.27-1.68)	0.39
Female	3	2.1 (0.96-4.63)	0.06

CI – confidence interval, n – number of studies, OR – odds ratio

### Associated factors for OD in hospitalized COVID-19 patients

The study findings demonstrated that intubation, tracheostomy use, and proning were significantly associated with OD, while mechanical ventilation, gender, and comorbidities including respiratory diseases, hypertension, diabetes mellitus, and neurological diseases were not. Regarding intubation, intubated COVID-19 patients were more likely to develop OD compared to non-intubated COVID-19 patients (OR = 16.3; 95% CI = 7.10, 37.3; high certainty of evidence). COVID-19 patients who were managed with tracheostomies were at higher risk of developing OD compared to COVID-19 patients with no tracheostomies (OR = 8.09; 95% CI = 3.05, 21.5; high certainty of evidence). COVID-19 patients who were managed in a prone position were at higher risk of developing OD compared to COVID-19 patients who were not managed in prone position (OR = 4.97, 95% CI = 1.34, 18.5; high certainty of evidence). COVID-19 patients who were mechanically ventilated were not associated with risk of developing OD (OR = 3.32; 95% CI = 0.30-36.7; low certainty of evidence). Regarding comorbidities, respiratory diseases (OR = 2.41; 95% CI = 0.52, 11.1; low certainty of evidence), hypertension (OR = 2.06; 95% CI = 0.45, 9.58; low certainty of evidence), diabetes mellitus (OR = 1.29; 95% CI = 0.62, 2.66; moderate certainty of evidence), and neurological diseases (OR = 1.27; 95% CI = 0.14, 11.6; low certainty of evidence) were not associated with risk of developing OD. Regarding gender, being female (OR = 2.11; 95% CI = 0.96, 4.63; moderate certainty of evidence) and being male (OR = 0.67; 95% CI = 0.27, 1.68; moderate certainty of evidence) was not associated with risk of developing OD (**Table 4**).

### Results of the moderator analysis for OD

The results of the moderator analysis for prevalence of OD showed that setting, ventilation status, intubation status, and assessment tool were significant moderator variables, while age, male and female percentage, proning status, neurologic comorbidities, study quality, study design, and study type were not significant moderator variables. Regarding the ventilation status (*P* = 0.01), studies that used COVID-19 patients who were mechanically ventilated had an OD prevalence of 71% compared to a prevalence of 25% for studies with non-ventilated COVID-19 patients and a prevalence of 15% for studies that had combined ventilated and non-ventilated COVID-19 patients. Regarding the intubation status (*P* = 0.01), studies that used COVID-19 patients who were post-intubated had an OD prevalence of 63% compared to a prevalence of 18% for studies with non-intubated COVID-19 patients and a prevalence of 16% for studies that had combined ventilated and non-ventilated COVID-19 patients. Regarding assessment tools used (*P* = 0.01), studies that used invasive assessment methods had a prevalence of 90% compared to a prevalence of 37% for studies that used non-invasive objective assessment tool and a prevalence of 29% for studies that used non-invasive assessment method. Age (*P* = 0.29) demonstrated a 7% non-significant increase in the prevalence of OD among hospitalized COVID-19 patients. We found no significant gender differences, with a 3% increase for males (*P* = 0.12) and 3% decrease for females (*P* = 0.29) in the prevalence of OD among hospitalized COVID-19 patients. Regarding neurologic comorbidities (*P* = 0.93), studies that used hospitalized COVID-19 patients with neurologic comorbidities had a prevalence of 36% compared to a prevalence of 34% for studies that used hospitalized COVID-19 patients without neurologic comorbidities. Regarding study quality (*P* = 0.14), poor-quality studies had a prevalence of 55% compared to 43% for moderate-quality studies and 21% for high-quality studies. Regarding study design (*P* = 0.95), studies that used retrospective designs had a prevalence of 36% compared to a prevalence of 35% for studies that used prospective designs. Regarding study type (*P* = 0.80), document review studies had a prevalence of 38% compared to a prevalence of 33% for primary studies **(****Table 3****)**.

## DISCUSSION

### Prevalence of OD and risk of mortality among COVID-19 patients

To our knowledge, this is the first meta-analysis to provide comprehensive evidence on the prevalence of OD and risk of mortality among COVID-19 patients. We found a substantial prevalence of OD estimated at 35%, with a 6.41 times risk of mortality among hospitalized COVID-19 patients. Previous study findings have shown a higher prevalence rate of OD in post-extubated critically ill patients, acute stroke patients, and older adults, with prevalence rates being closer to 50% [4,5,34]. McIntyre et al. [34] found an incidence rate of 41% for OD among hospitalized critically-ill patients secondary to intubation, while the prevalence of OD has been estimated at 42% among acute stroke patients [4] and 46% among older adults [5]. Previous evidence suggests that COVID-19 virus invasion of the central and peripheral nervous system leads to damage of the swallowing neural control and causes impaired oropharyngeal sensory function, leading to oropharyngeal dysphagia [6]. This meta-analysis showed that the observed OD prevalence was higher in COVID-19 patients who were mechanically ventilated, post-intubated, and admitted to an ICU. The possible explanation for the higher prevalence of OD is that the management of acute SARS secondary to COVID-19 with intubation and mechanical ventilation likely led to the observed higher rates, as they have been shown to cause damage to oropharyngeal structures, leading to swallowing impairment in critically-ill patients without COVID-19. Nevertheless, the pathway of the COVID-19 virus’ neurological involvement requires further investigation to improve our understanding of the development of OD in this population.

Most of the included studies in this meta-analysis used non-invasive subjective tools among hospitalized COVID-19 patients, which may have likely led to the underestimation of OD. Considering that the COVID-19 virus is highly infectious and contagious, the assessment of OD using invasive objective tools being the gold standard would pose a great risk in the spread of the virus. As such, the use of invasive objective tools in hospitalized COVID-19 patients should be done with caution and deserves utmost and careful consideration. Our meta-analysis also found that hospitalized COVID-19 patients with OD had a higher risk of mortality compared to those without OD. OD predisposes to aspiration, leading to aspiration pneumonia, and the COVID-19 virus causes severe respiratory distress syndrome, predisposing those COVID-19 patients with OD even at higher risk of mortality [4,5]. Thus, an early assessment of OD in hospitalized, mechanically ventilated, and post-intubated COVID-19 patients admitted to an ICU is key to early detection of aspiration, leading to early prevention and management of aspiration pneumonia and a reduced risk of mortality.

### Factors associated with OD in hospitalized COVID-19 patients

Intubation, tracheostomy use, and proning were factors significantly associated with OD among hospitalized COVID-19 patients. Intubated COVID-19 patients were more likely to develop OD compared to non-intubated COVID-19 patients. Endotracheal intubation is linked with OD due to oropharyngeal and laryngeal damage, altered sensory sensations, gastroesophageal reflux, neuromuscular weakness, reduced laryngeal sensitivity, and impaired synchronization of breathing and swallowing [7,35]. Additionally, the duration of the intubation and reduced lung function due to COVID-19 SARS further affect the swallowing process. COVID-19 patients who were tracheostomized were more likely to develop OD compared to COVID-19 patients who were not tracheostomized. Tracheostomy has been shown to cause decreased sensory input, reduced subglottic air pressure, and disuse atrophy of laryngeal structures, leading to OD [36]. COVID-19 patients who were managed in a prone position were more likely to develop OD compared to COVID-19 patients who were not managed in a prone position. Prolonged prone position predisposes COVID-19 patients to aspiration of secretions and microorganisms due to relaxed oropharyngeal and laryngeal muscles as a result of sedation while being mechanically ventilated [7,37]. However, intubation, use of tracheostomy, and proning are established risk factors for OD and may likely confound the association between COVID-19 virus neurological involvement and subsequent development of OD in this population. Future studies exploring the association between COVID-19 virus neurological involvement and OD should adjust and pay attention to these risk factors. Nevertheless, efforts to ensure early assessment of OD in COVID-19 patients who have been post-intubated, tracheostomised, and nursed in a prone position requires greater attention. Furthermore, we found that mechanical ventilation, gender, and comorbidities (including respiratory diseases, hypertension, diabetes mellitus, and neurological diseases) were not significant factors for OD in this population. As few studies reported detailed demographic characteristics of hospitalized COVID-19 patients with OD, the association of these demographic characteristics with OD may either be under-estimated or over-estimated. Therefore, future prospective studies with detailed and comprehensive adjusted demographic characteristics for hospitalized COVID-19 patients are encouraged to address and clarify this shortfall.

### Study strengths and limitations

This meta-analysis has several strengths. It is the first to provide comprehensive evidence on the prevalence of OD and risk of mortality among hospitalized COVID-19 patients. Second, we conducted a comprehensive search to identify includable studies without any language limitations. Third, we followed the MOOSE and PRISMA guidelines in the conduct and reporting and registered the study protocol in PROSPERO to ensure research integrity and transparency. Fourth, we examined the included studies’ quality using Hoy’s risk of bias assessment tool for prevalence studies and determined the certainty of evidence of the included outcomes, which ranged from low to high. However, there are some limitations in the interpretation of the results. First, we observed statistical heterogeneity and used meta-regression and sub-group analyses to determine and identify moderator variables to explain the sources of the heterogeneity. Second, due to limited demographic characteristics, the association of the analysed demographic characteristics of the hospitalized COVID-19 patients with OD may be under-estimated or over-estimated and detailed demographic characteristics are needed in future studies to address this issue. Lastly, the generalizability of our findings is limited to hospitalized COVID-19 patients; future studies should explore the prevalence of COVID-19-associated OD in other settings, including the community.

## CONCLUSIONS

This meta-analysis shows a substantial OD prevalence and a high mortality risk among hospitalized COVID-19 patients. Additionally, intubation, tracheostomy use, and proning were associated with higher risk of developing OD. We found that the prevalence of OD was higher among COVID-19 patients who were admitted to ICU, intubated, and mechanically ventilated. As such, early assessment of OD in hospitalized COVID-19 patients should be necessary to ensure early detection of aspiration, leading to early prevention and management of aspiration pneumonia and reduced risk of mortality. Future studies with detailed and comprehensive adjusted demographic characteristics for hospitalized COVID-19 patients are needed to address and clarify the association between COVID-19 virus neurological involvement and OD.

## Additional material


Online Supplementary Document
