# Supplementary material for: Prevalence of oropharyngeal dysphagia and risk of mortality among hospitalized COVID-19 patients: A meta-analysis
Source: J Glob Health. 2022 Dec 29;12:05058. doi: 10.7189/jogh.12.05058 (PMC9798345; doi:10.7189/jogh.12.05058)

Online Supplementary Document

Table S1: Search Strategy……………………….…………………………………….……2

Table S2: Risk of Bias Assessment…………………………..………………..…………5

Table S3: Certainty of Evidence….…………………………..…………………..…………6

Figure S1: Funnel Plot for Publication Bias…………………....…………………..………7

Table S1: Search Strategy

Database	Mesh-Terms	Number of studies
CINAHL	(prevalence OR incidence OR epidemiology OR rate OR rates OR number OR proportion OR probability OR event) AND (dysphagia OR swallowing disorder OR deglutition disorder OR oropharyngeal dysphagia) AND (COVID-19 or covid-19 OR Corona virus OR SARS-COV 2) in Title Abstract Keyword	29
Embase	('prevalence'/exp OR prevalence OR 'incidence'/exp OR incidence OR 'epidemiology'/exp OR epidemiology OR rate OR rates OR 'number'/exp OR number OR proportion OR 'probability'/exp OR probability OR event) AND ('dysphagia'/exp OR dysphagia OR 'swallowing disorder'/exp OR 'swallowing disorder' OR (('swallowing'/exp OR swallowing) AND ('disorder'/exp OR disorder)) OR 'deglutition disorder'/exp OR 'deglutition disorder' OR (('deglutition'/exp OR deglutition) AND ('disorder'/exp OR disorder)) OR 'oropharyngeal dysphagia'/exp OR 'oropharyngeal dysphagia' OR (oropharyngeal AND ('dysphagia'/exp OR dysphagia))) AND ('covid 19'/exp OR 'covid 19' OR 'corona virus'/exp OR 'corona virus' OR (corona AND ('virus'/exp OR virus)) OR 'sars-cov 2'/exp OR 'sars-cov 2' OR (('sars cov'/exp OR 'sars cov') AND ('2'/exp OR 2)))	554
PubMed	("epidemiology"[MeSH Subheading] OR "epidemiology"[All Fields] OR "prevalence"[All Fields] OR "prevalence"[MeSH Terms] OR "prevalance"[All Fields] OR "prevalences"[All Fields] OR "prevalence s"[All Fields] OR "prevalent"[All Fields] OR "prevalently"[All Fields] OR "prevalents"[All Fields] OR ("epidemiology"[MeSH Subheading] OR "epidemiology"[All Fields] OR "incidence"[All Fields] OR "incidence"[MeSH Terms] OR "incidences"[All Fields] OR "incident"[All Fields] OR "incidents"[All Fields]) OR ("epidemiologies"[All Fields] OR "epidemiology"[MeSH Subheading] OR "epidemiology"[All Fields] OR "epidemiology"[MeSH Terms] OR "epidemiology s"[All Fields]) OR ("j rehabil assist technol eng"[Journal] OR "rate"[All Fields]) OR "rates"[All Fields] OR ("number"[All Fields] OR "numbers"[All Fields]) OR ("proportion"[All Fields] OR "proportions"[All Fields]) OR ("probability"[MeSH Terms] OR "probability"[All Fields] OR "probabilities"[All Fields]) OR ("event"[All Fields] OR "event s"[All Fields] OR "events"[All Fields])) AND ("deglutition disorders"[MeSH Terms] OR ("deglutition"[All Fields] AND "disorders"[All Fields]) OR "deglutition disorders"[All Fields] OR "dysphagia"[All Fields] OR "dysphagias"[All Fields] OR ("deglutition disorders"[MeSH Terms] OR ("deglutition"[All	118

	Fields] AND "disorders"[All Fields]) OR "deglutition disorders"[All Fields] OR ("swallowing"[All Fields] AND "disorder"[All Fields]) OR "swallowing disorder"[All Fields]) OR ("deglutition disorders"[MeSH Terms] OR ("deglutition"[All Fields] AND "disorders"[All Fields]) OR "deglutition disorders"[All Fields] OR ("deglutition"[All Fields] AND "disorder"[All Fields]) OR "deglutition disorder"[All Fields]) OR ("deglutition disorders"[MeSH Terms] OR ("deglutition"[All Fields] AND "disorders"[All Fields]) OR "deglutition disorders"[All Fields] OR ("oropharyngeal"[All Fields] AND "dysphagia"[All Fields]) OR "oropharyngeal dysphagia"[All Fields])) AND ("covid 19"[All Fields] OR "covid 19"[MeSH Terms] OR "covid 19 vaccines"[All Fields] OR "covid 19 vaccines"[MeSH Terms] OR "covid 19 serotherapy"[All Fields] OR "covid 19 serotherapy"[Supplementary Concept] OR "covid 19 nucleic acid testing"[All Fields] OR "covid 19 nucleic acid testing"[MeSH Terms] OR "covid 19 serological testing"[All Fields] OR "covid 19 serological testing"[MeSH Terms] OR "covid 19 testing"[All Fields] OR "covid 19 testing"[MeSH Terms] OR "sars cov 2"[All Fields] OR "sars cov 2"[MeSH Terms] OR "severe acute respiratory syndrome coronavirus 2"[All Fields] OR "ncov"[All Fields] OR "2019 ncov"[All Fields] OR (("coronavirus"[MeSH Terms] OR "coronavirus"[All Fields] OR "cov"[All Fields]) AND 2019/11/01:3000/12/31[Date - Publication]) OR ("covid 19"[All Fields] OR "covid 19"[MeSH Terms] OR "covid 19 vaccines"[All Fields] OR "covid 19 vaccines"[MeSH Terms] OR "covid 19 serotherapy"[All Fields] OR "covid 19 serotherapy"[Supplementary Concept] OR "covid 19 nucleic acid testing"[All Fields] OR "covid 19 nucleic acid testing"[MeSH Terms] OR "covid 19 serological testing"[All Fields] OR "covid 19 serological testing"[MeSH Terms] OR "covid 19 testing"[All Fields] OR "covid 19 testing"[MeSH Terms] OR "sars cov 2"[All Fields] OR "sars cov 2"[MeSH Terms] OR "severe acute respiratory syndrome coronavirus 2"[All Fields] OR "ncov"[All Fields] OR "2019 ncov"[All Fields] OR (("coronavirus"[MeSH Terms] OR "coronavirus"[All Fields] OR "cov"[All Fields]) AND 2019/11/01:3000/12/31[Date - Publication])) OR (("corona"[All Fields] OR "coronae"[All Fields] OR "coronas"[All Fields]) AND ("virology"[MeSH Subheading] OR "virology"[All Fields] OR "viruses"[All Fields] OR "viruses"[MeSH Terms] OR "virus s"[All Fields] OR "viruse"[All Fields] OR "virus"[All Fields])) OR ("sars cov	

	2"[MeSH Terms] OR "sars cov 2"[All Fields] OR "sars cov 2"[All Fields]))	
Scopus	(prevalence OR incidence OR epidemiology OR rate OR rates OR number OR proportion OR probability OR event) AND (dysphagia OR swallowing disorder OR deglutition disorder OR oropharyngeal dysphagia) AND (COVID-19 or covid-19 OR Corona virus OR SARS-COV 2)	59
Web of Science	prevalence OR incidence OR epidemiology OR rate OR rates OR number OR proportion OR probability OR event **(All Fields) and** dysphagia OR swallowing disorder OR deglutition disorder OR oropharyngeal dysphagia **(All Fields) and** COVID-19 or covid-19 OR Corona virus OR SARS-COV 2 **(All Fields)**<br>Timespan: All years. Indexes: SCI-EXPANDED, SSCI.	50
WHO COVID-19 Databse	(prevalence OR incidence OR epidemiology OR rate OR rates OR number OR proportion OR probability OR event) AND (dysphagia OR swallowing disorder OR deglutition disorder OR oropharyngeal dysphagia) AND (COVID-19 or covid-19 OR Corona virus OR SARS-COV 2)	100

Table S2: Risk of Bias Assessment

Study	External validity				Internal validity						Total
	Representativeness	Sampling frame	Random selection	Non-response	Data collection	Case definition	Instrument	Same mode	Prevalence period	Numerator / denominator	
Archer, 2021	1	1	0	1	1	1	1	1	1	1	9 – L
Ceruti, 2021	0	0	0	1	0	1	1	1	1	1	6 – H
Clayton, 2022	0	1	0	1	1	1	1	1	1	1	8 – M
Gonzalez Lindh, 2022	0	1	0	1	1	1	1	1	1	1	8 – M
Grilli, 2022	0	1	0	1	1	1	1	1	1	1	8 – M
Lagier, 2021	0	1	0	1	1	1	1	1	1	1	8 – M
Laguna, 2021	0	1	0	1	0	1	1	1	1	1	7 – M
Leis-Cofino, 2021	1	1	0	1	1	1	1	1	1	1	9 – L
Lima, 2020	1	1	0	1	0	1	1	1	1	1	8 – M
Marchese, 2022	1	1	0	1	1	1	1	1	1	1	9 – L
Martin-Martinez, 2021	1	1	0	1	1	1	1	1	1	1	9 – L
Olezene, 2021	0	0	0	1	0	1	1	1	1	1	6 – H
Regan, 2021	1	1	0	1	1	1	1	1	1	1	9 – L
Reyes-Torres, 2021	1	1	0	1	1	1	1	1	1	1	9 – L
Rouhani, 2021	0	1	0	1	1	1	1	1	1	0	7 – M
Webler, 2022	0	0	0	1	0	1	1	1	1	1	6 – H
Yamada, 2022	1	1	0	1	0	1	1	1	1	0	7 – M
Yilmaz, 2021	1	1	0	1	0	1	1	1	1	1	8 – M

Score of 1 for low risk and 0 for high risk. Poor quality: score of ≤ 6 (H), moderate quality: score of 7 or 8 (M), high quality: score of 9 or 10 (L)

Table S3: Certainty of Evidence

Variables	Risk of Bias	Indirectness	Imprecision	Publication Bias	Inconsistency	Certainty of Evidence
**Pooled Prevalence**	Serious	Not serious	Not serious	Not serious	Serious	Low ⊕⊕
**Associated Outcome**						
Mortality	Not serious	Not serious	Not serious	Not serious	Serious	Moderate ⊕⊕⊕
**Associated Factors**						
Intubation	Not serious	Not serious	Not serious	Not serious	Not serious	High ⊕⊕⊕⊕
Tracheostomy use	Not serious	Not serious	Not serious	Not serious	Not serious	High ⊕⊕⊕⊕
Proning	Not serious	Not serious	Not serious	Not serious	Not serious	High ⊕⊕⊕⊕
Mechanical ventilation	Not serious	Not serious	Serious	Not serious	Serious	Low ⊕⊕
**Comorbidities**						
Respiratory disease	Not serious	Not serious	Serious	Not serious	Serious	Low ⊕⊕
Hypertension	Not serious	Not serious	Serious	Not serious	Serious	Low ⊕⊕
Diabetes Mellitus	Not serious	Not serious	Serious	Not serious	Not serious	Moderate ⊕⊕⊕
Neurological disease	Not serious	Not serious	Serious	Not serious	Serious	Low ⊕⊕
**Participant's characteristics**						
Male	Not serious	Not serious	Serious	Not serious	Not serious	Moderate ⊕⊕⊕
Female	Not serious	Not serious	Serious	Not serious	Not serious	Moderate ⊕⊕⊕

Figure S1: Funnel Plot for Publication Bias

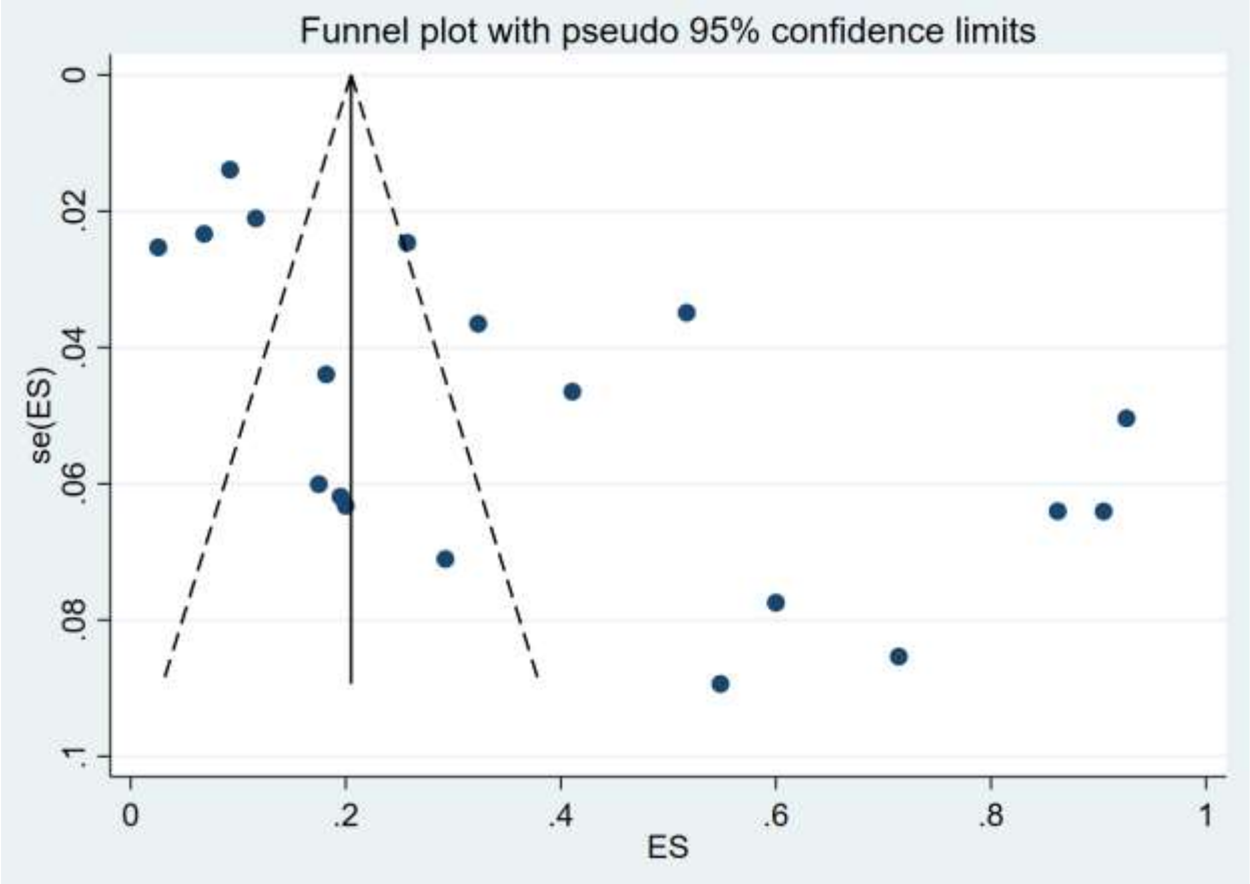

Supplement: Online Supplementary Document [file jogh-12-05058-s001.pdf]
